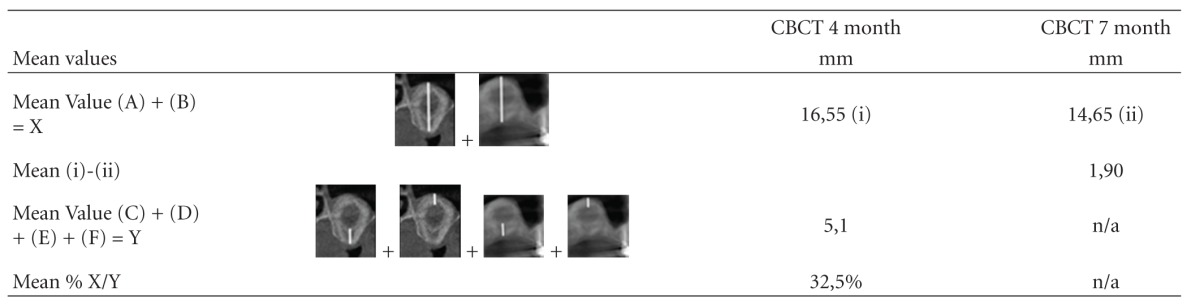# Biological Principles and Physiology of Bone Regeneration under the Schneiderian Membrane after Sinus Lift Surgery: A Radiological Study in 14 Patients Treated with the Transcrestal Hydrodynamic Ultrasonic Cavitational Sinus Lift (Intralift)

**DOI:** 10.1155/2012/576238

**Published:** 2012-06-17

**Authors:** A. Troedhan, A. Kurrek, M. Wainwright

**Affiliations:** ^1^Center for Facial Esthetics Vienna, Brauhausgasse 12-14, 1050 Vienna, Austria; ^2^Implantology Clinic Ratingen, Lintorfer Straße 7, 40878 Ratingen, Germany; ^3^Implantology Clinic Kaiserswerth, Kaiserswerther Markt 25, 40489 Düsseldorf, Germany

## Abstract

*Introduction*. Sinus lift procedures are a commonly accepted method of bone augmentation in the lateral maxilla with clinically good results. Nevertheless the role of the Schneiderian membrane in the bone-reformation process is discussed controversially. Aim of this study was to prove the key role of the sinus membrane in bone reformation in vivo. *Material and Methods*. 14 patients were treated with the minimal invasive tHUCSL-Intralift, and 2 ccm collagenous sponges were inserted subantrally and the calcification process followed up with CBCT scans 4 and 7 months after surgery. *Results*. An even and circular centripetal calcification under the sinus membrane and the antral floor was detected 4 months after surgery covering 30% of the entire augmentation width/height/depth at each wall. The calcification process was completed in the entire augmentation volume after 7 months. A loss of approximately 13% of absolute augmentation height was detected between the 4th and 7th month. *Discussion*. The results of this paper prove the key role of the sinus membrane as the main carrier of bone reformation after sinus lift procedures as multiple experimental studies suggested. Thus the importance of minimal invasive and rupture free sinuslift procedures is underlined and does not depend on the type of grafting material used.

## 1. Introduction

Although subantral augmentation procedures (Sinus lifting) can be considered as an established and highly successful method to multiply bone prior to implant insertion into the lateral maxilla site, the biological mechanisms of subantral bone regeneration are still focus of controversial scientific discussions.

While in the eighties and nineties of the past century the discussion on graft material inserted subantrally focused on free autologous bone grafts the mainstream research turned over to heterologous, allogenic, xenogenic and synthetic bone graft materials.

Concerning free autologous bone grafts most questions were already answered in the late sixties of the past century by Scandinavian scientists.

Puranen [1] proved free autologous bone grafts stored in room air to lose all biological activity within 90 minutes, when kept in saline solution within 3 hours. Bohr et al. [2] investigated the osteogenic potency of freshly harvested autologous bone grafts in comparison to deproteinized cadaver bone: although he reported a better reossification of the fresh free autologous transplants in the augmentation site in the first five days following surgery, the overall advantage of fresh autologous bone grafts was beyond any experimental and clinical significance after the standard healing period.

The key role of the periosteum in bone healing and regeneration was proven in other disciplines of medicine for quite a time [3–5] and was verified again only lately [6, 7] but mostly neglected in dentistry and oral surgery.

Lundgren et al. [8] 2004 found sufficient bone regeneration after Sinus lift surgery without the insertion of any bone graft material but sufficient bleeding into the subantral space but left open the answer to the question about the regeneration mechanisms which were then published by Srouji et al. in 2009 [9, 10]: the basal cell layer of the Schneiderian membrane is periosteum—as any other membrane covering vital bone like the Dura mater [5, 6]—that solely produces all necessary cellular and humoral factors for bone healing and bone regeneration such as Bone Morphing protein 2 (which has a key function in bone regeneration [11]), osteonectin, osteocalcin, and osteopontin.

Vital periosteum alone initiates bone regeneration and production in absence of any calcified structure or the presence of osteocytes needing only a stable blood coagulum as Srouji et al. were able to prove [10].

Based on the knowledge of the superior atraumaticity of ultrasonic surgery [12, 13] and of bone regeneration mechanisms under the Schneiderian membrane and the mandatory atraumatic detachment of the sinus membrane from the antral bone, the authors (TKW-Research-Group) developed the minimal invasive transcrestal hydrodynamic ultrasonic cavitational Sinus lift (tHUCSL-Intralift) for Piezotome I/II/SOLO in cooperation with Satelec-ACTEON/France to preserve the sinus-membrane and its key function in the later bone regeneration [14–17].

The aim of the present study was to verify in vivo the postulated bone regeneration capabilities of the periosteum of the Schneiderian membrane in patients treated with the tHUCSL-Intralift by detecting the origins of the calcification process radiographically on macroscopical level.

## 2. Material and Methods

Within a multicenter study on the success rates of the tHUCSL-Intralift using various radiopaque bone graft materials for subantral augmentation, 14 patients (8 female, 6 male) at an average age of 52 yrs (±16 yrs) were selected with vastly pneumatized sinuses on the right side and remaining subantral alveolar crest heights of 4 mm or less. Instead of radiopaque bone graft material only, a radiolucid collagenous sponge of a stable and defined volume of approximately 2 ccm was inserted subantrally to radiographically follow up the origins of new bone growth and calcification processes in CBCT scans to indirectly verify the findings by Lundgren et al. [8] and Srouji et al. [9, 10] in human sinuses in vivo.

Sinus lift surgery on the right maxillary sinus was performed according to the strict tHUCSL-Intralift protocol.

**Figure 1:**
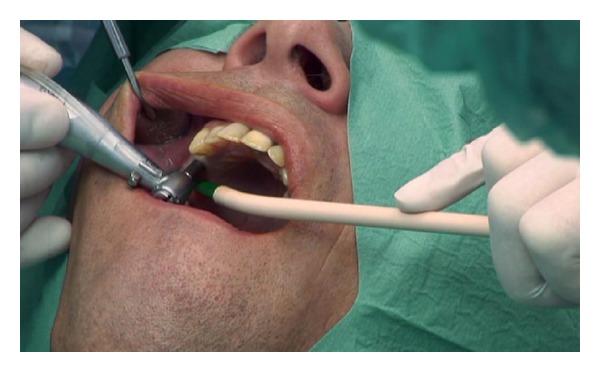
Intralift: 6 mm gingival punch or 6 × 6 mm top crestal flap to approach the alveolar crest.

**Figure 2:**
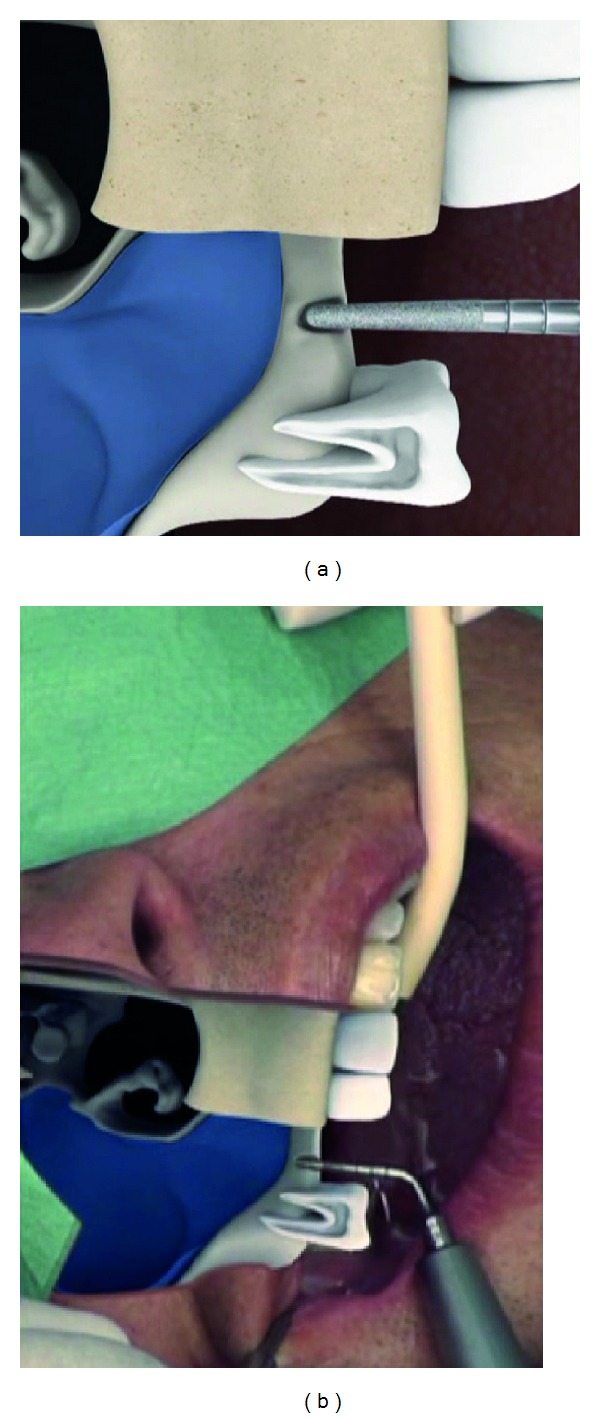
Intralift: trepanation of the subantral alveolar crest with the conical diamond coated tip TKW 1 for Piezotome.

The subantral alveolar crest was revealed by either a single or dual 6 mm diameter gingival punch or an 6 mm rectangular top crestal mucoperiosteal flap (Figure 1). A pilot trepanation was performed with the diamond-coated TKW 1 ultrasonic tip for Piezotome I/II/SOLO (Satelec-ACTEON/France) (Figure 2).

**Figure 3:**
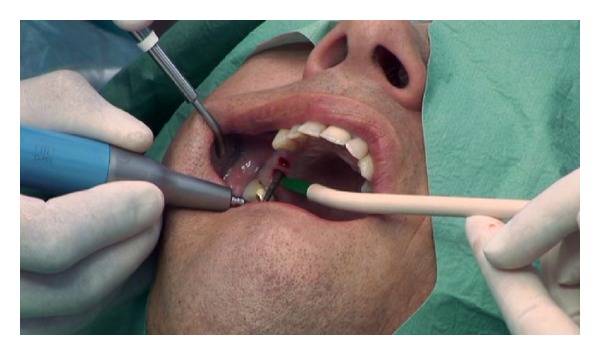
Intralift: opening of the sinus floor with the round diamond coated tip TKW2 for Piezotome.

**Figure 4:**
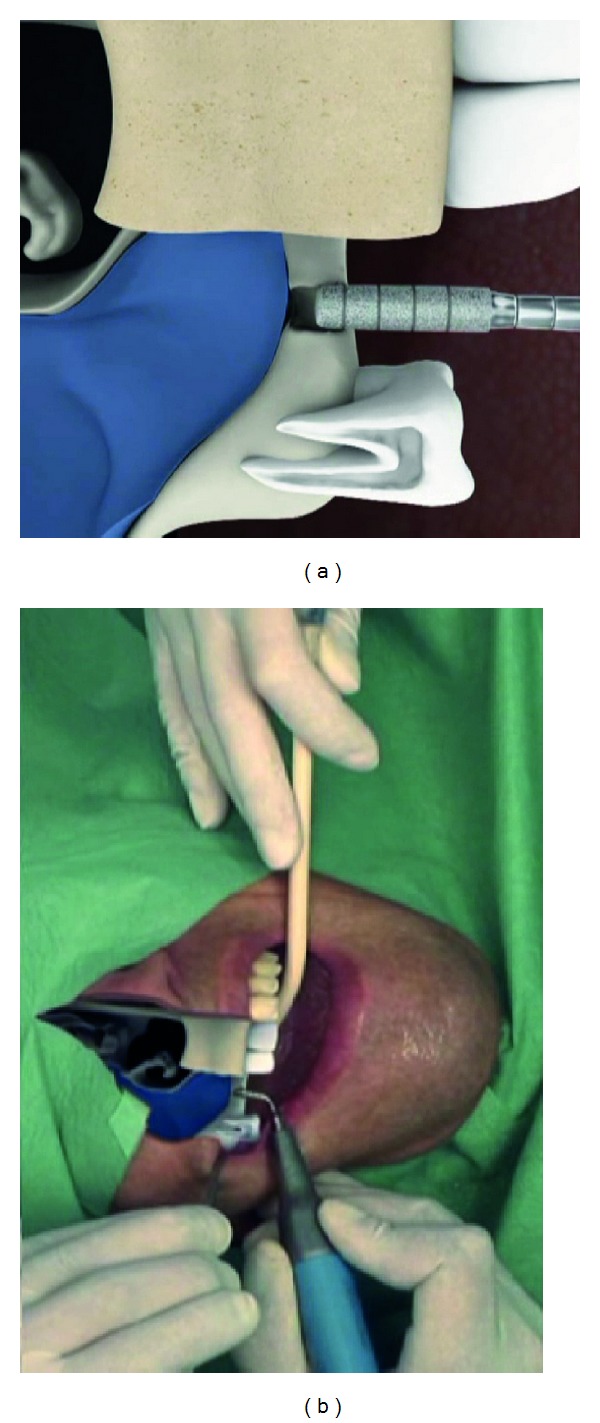
Intralift: preparation of the receptacle for the hydrodynamic cavitational ultrasound applicator with the diamond-coated tip TKW4 for Piezotome (preparation of a ventile seat).

The sinus floor was opened with the diamond-coated atraumatic TKW 2-ultrasonic tip (Figure 3) followed by the preparation of a receptacle for the elevation applicator TKW 5 with the flat diamond-coated TKW 4 ultrasonic tip (Figure 4).

**Figure 5:**
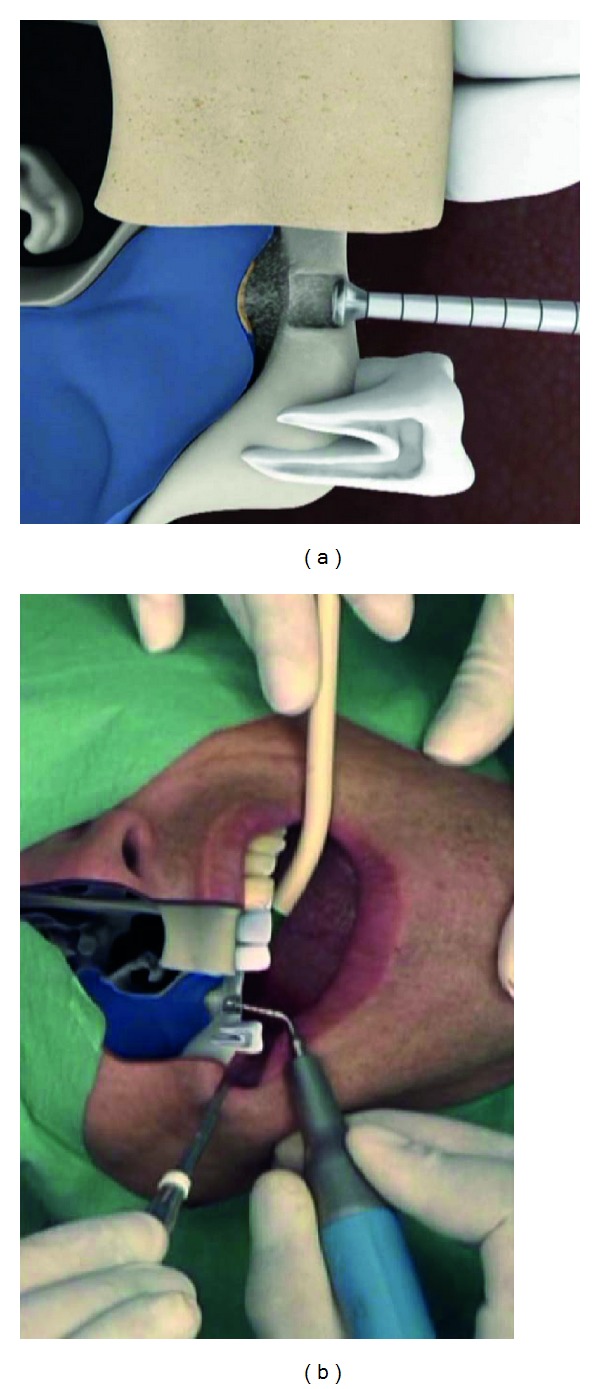
Intralift: detachment of the sinus membrane with the detachment applicator TKW5 which is sealed towards the oral cavity by the receptacle. By hydrodynamic cavitational pressure the sinus membrane is elevated and a subantral volume of 2,5 ccm created.

The sinus membrane then was atraumatically separated from the antral bone with the hydrodynamic ultrasonic cavitational applicator TKW 5 (Figure 5) at a flow rate of saline solution of 30 mL/min for 5 seconds thus creating a subantral volume of 2,5 ccm under the elevated sinus membrane. (Although the differences in physics between a hydraulic and a hydrodynamic cavitational separation of the sinus membrane from the bone are significant, the basic process can be circumscribed as detaching and elevating the membrane with water-pressure).

**Figure 6:**
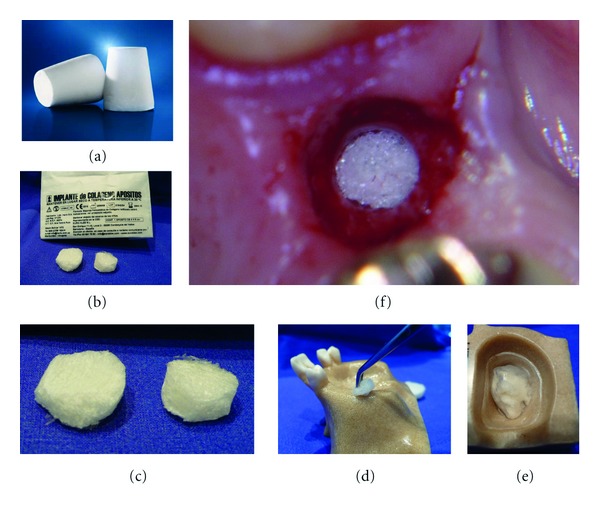
Collagenous sponges used: (a) Resorba Dentalkegel/Resorba/GER (1,9 ccm), ((b), (c)) Implante Colageno/EURO-Klee/ES (2,0 ccm), (d) insertion demonstration on a training model (the sponge is inserted after the sinus-membrane was elevated with the Intralift method), (e) view from inside the sinus in a training model, (f) surgical site with sponge inserted.

Once the elevated sinus-membrane was verified to float free and unperforated/unruptured in the traditional unilateral Valsalva check, a form stable radiolucent collagenous sponge of approximately 2 ccm (Implante Colageno/EURO-Klee/Spain or Parasorb-Dentalkegel/RESORBA/Germany, (Figures 6(a)–6(e)) was inserted subantrally instead of radiopaque bone graft material to stabilize the elevated sinus membrane as well as the blood clot forming underneath and maintain the elevation volume achieved with the tHUCSL-Intralift procedure. Patients were followed up for pain, swelling, and any sign of nightly bleeding out of the corresponding nostril and/or observation of blood-contaminated sputum and/or unusual sneezing attacks one, two, three, and saven days after surgery. Implants were inserted into the augmented site 8 months after tHUCSL-Intralift and prosthodontic treatment latest completed 11-12 months after initial Intralift surgery.

**Figure 7:**
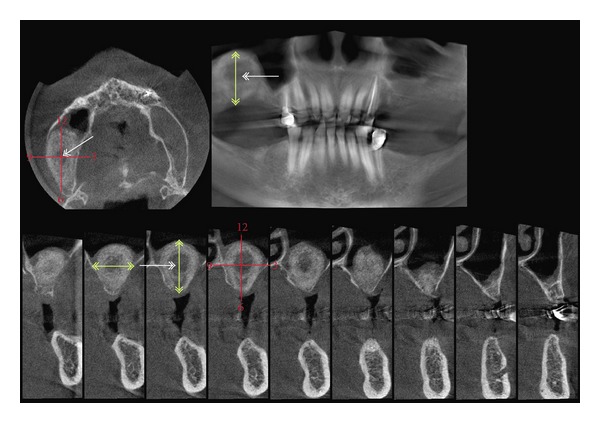
CBCT scan measurements: yellow arrows: total distances height/width/depth, red reference cross: measurements of calcification thicknesses in 3, 6, 9, and 12 o'clock position.

Radiographic followup was performed 4 and 7 months following surgery with calibrated CBCT scans and the scans modulated with sharpness, edge detection and contrast filters as well as additive and subtractive grayscale enhancement filters for better distinction between soft and hard tissues. The calcification process was determined with grayscale match algorithms to the surrounding natural bone in mm in the augmentation area with the augmentation center as origin (Figure 7 white arrow) in transversal, sagittal, and horizontal scan slides with the calibrated CBCTs measurement tool.

 Measurements were taken in mm measuring the absolute height of the augmentation including the alveolar crest in transversal and sagittal slides (Figure 7 yellow arrow) and in 3, 6, 9, and 12 o'clock position (Figure 7 red reference cross) centripetally from the outer line of the visible calcification to the center. The maximum vertical height of the augmentation site was measured in the transversal and sagittal slides including the alveolar crest since a precise radiological separation of the newly formed bone from the remaining alveolar crest was not possible. The same procedure was applied to all measurements in 6 o'clock position.

## 3. Results

All 14 tHUCSL Intralift Sinus lift procedures were conducted without perforation of the sinus membrane, and no postsurgical complications suspicious of sinus-membrane perforations occurred. The mean height of the alveolar crest in the 14 study patients was 3,2 mm (st. dev. ± 0,8 mm) at the entrance site of the Intralift procedure measured intraoperatively.

**Figure 8:**
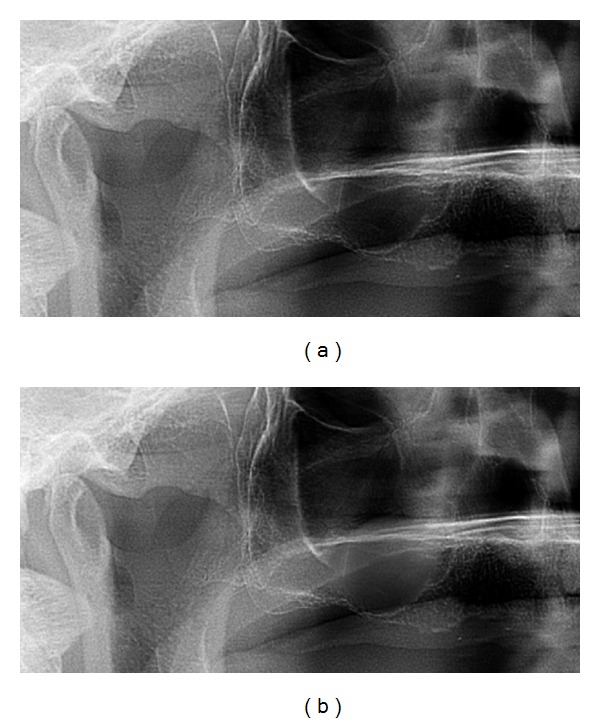
Immediate presurgical (a) and postsurgical (b) OPG: the collagenous sponge shows similar to a mucocele or less.

Figure 8 shows a typical presurgical (Figure 8(a)) and immediate postsurgical (Figure 8(b)) panoramic X-ray of a female study patient. In most cases the inserted sponge was similar to a typical mucocele or was not detectable at all in panoramic X-rays.

CBCT scans after 4 months revealed an average achieved augmentation height of 16,3 mm in the transversal slides (st. dev. 2,2 mm) and 16,8 mm in the sagittal slide (st.dev. 2,6 mm) which was reduced to an average of 14,6 mm in the transversal slides and 14,7 mm in the sagittal slides after 7 months (Table 1).

**Figure 9:**
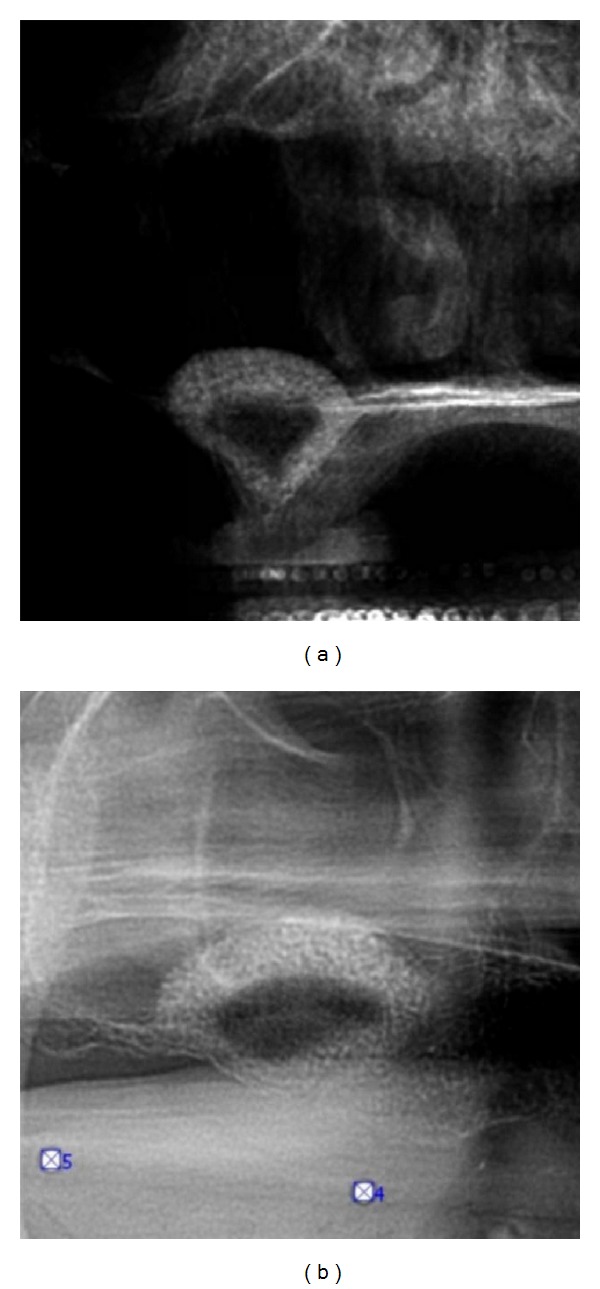
Transversal and parasagittal CBCT scan 4 months subsequent to tHUCSL-Intralift. The even circular centripetal calcification process can be observed.

The calcification process under the sinus-membrane radiologically showed an even centripetal circular distribution under the sinus-membrane and on the antral bone base with calcified tissue thicknesses of 3,6 mm to 4,3 mm (excluding all measurements in 6 o'clock position since these measurements include the original alveolar crest height) (Table 2, Figure 9).

**Figure 10:**
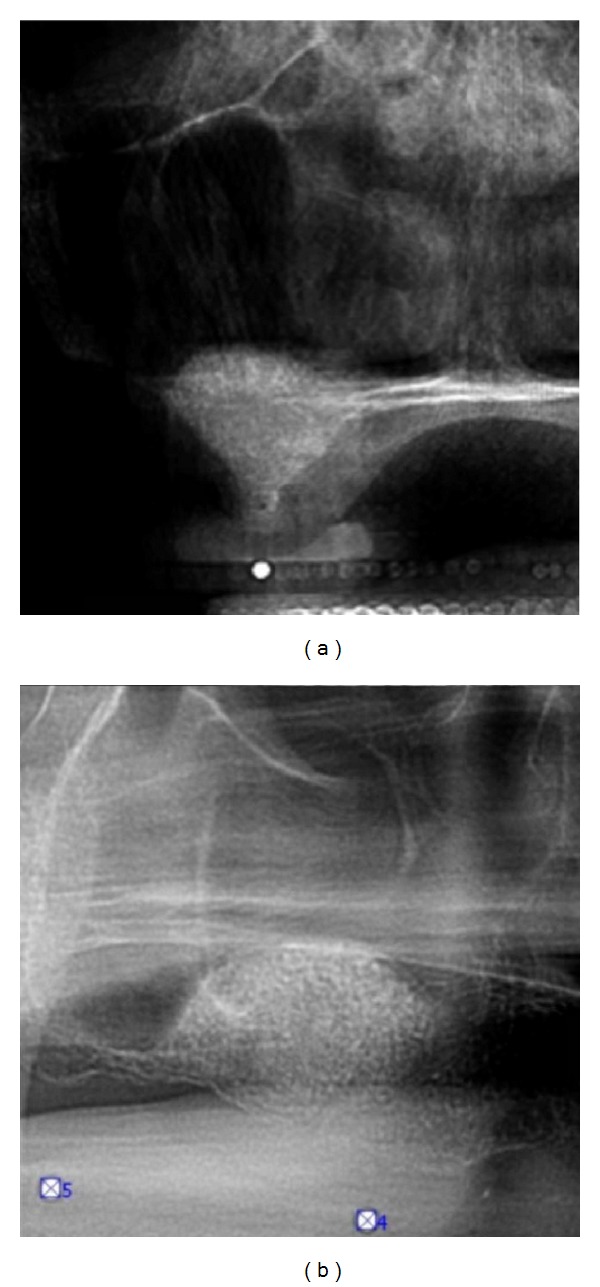
Transversal and parasagittal CBCT-scan 7 months post tHUCSL-Intralift. The ossification process is obviously completed.

After a healing period of 7 months all CBCT scans showed a completion of the calcification process in the augmented subantral volume except some randomly distributed minor radiolucent spots/areas thus not allowing a precise distinction for measurement between noncalcified areas and calcified tissue (Table 2, Figure 10).

The mean loss of absolute augmentation height of calcified tissue in the CBCT scans between 4 months and 7 months after surgery was 1,9 mm resulting in a final mean overall height of calcified tissue for implant insertion of 14,65 mm (Table 3).

After 4 month approximately a third of the subantral augmented volume in each measurement position (3, 6, 9, 12 o'clock) related to the total width/height/depth of the augmentation was presented as calcified tissue in the CBCT scans (Table 3, Figure 9). No precise distinction between calcified and noncalcified tissue could be made in the CBCT scans after 7 months.

**Figure 11:**
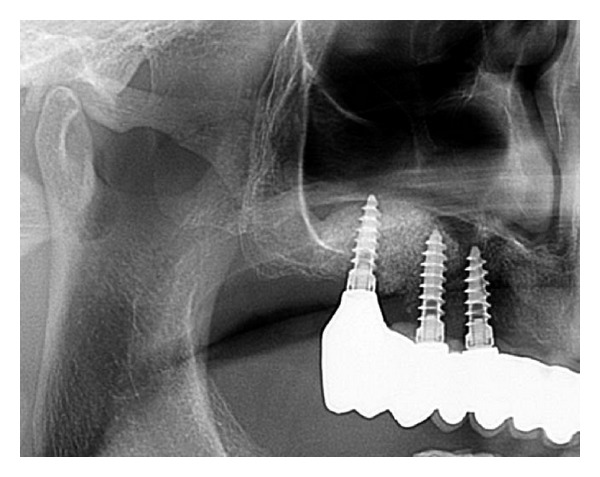
OPG with final prosthetic treatment after 11 months.

All patients were successfully treated with two-stage dental implants from various manufacturers (mostly Q2-Implant/TRINON Karlsruhe GmbH/Germany, BEGO RI/BEGO/Germany, SICace/SIC-Group/Germany and others) after 8 months and prosthetic suprastructure after 11-12 months (Figure 11).

Figures 12, 13, 14, 15, 16, 17, 18, 19, and 20 show two more typical cases of the present study.

## 4. Discussion

The radiological results of the present study confirm the experimental results published by Ortak et al. [7], Lundgren et al. [8], and Srouji et al. [9, 10] in vivo and suggest the Schneiderian membrane to be the primary carrier of bone reformation in Sinus lift procedures providing the necessary osteoprogenitor cells and humoral factors for bone regeneration [9, 11].

Nevertheless a volume stable subantral filling material is needed to stabilize the detached sinus membrane and formation of a blood coagulum in the upmost position to achieve sufficient augmentation heights and widths for implant insertion but the success of Sinus lift procedures does not seem to depend on the type of augmentation material (autologous, heterologous, xenogenic, synthetic calcified bone grafts) used. The results of this study proved a form stable collagenous sponge to be sufficient in stabilizing the sinus-membrane above the achieved subantral augmentation volume as well as the resulting stable blood clot forming in the collagenous sponge.

**Figure 17:**
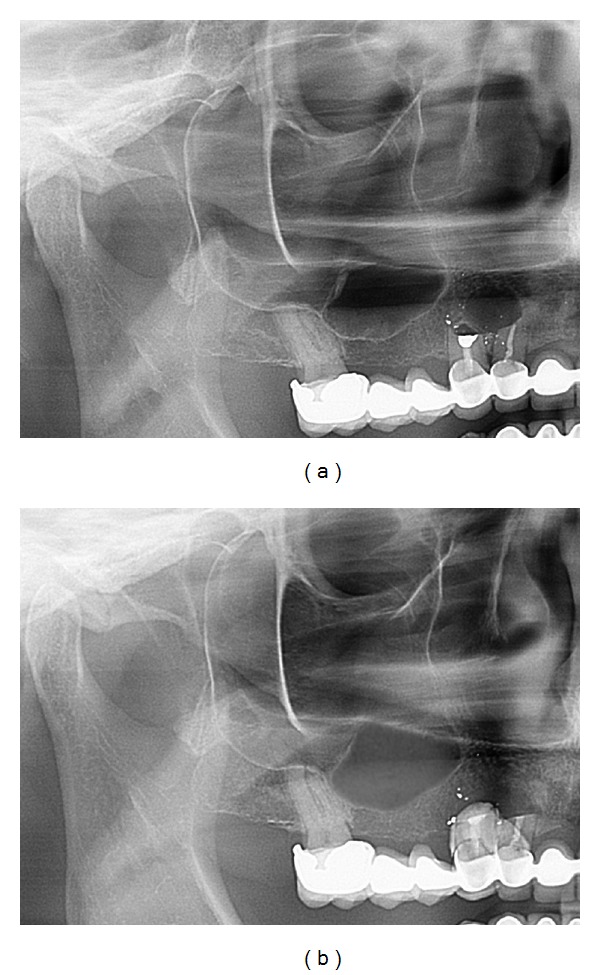
Case 3: presurgical (a) and immediate postsurgical (b) OPG: the collagenous sponge is almost not detectable. In this case the tHUCSL-Intralift was performed paracrestally from the buccal side due to the insufficient old bridge in site.

**Figure 18:**
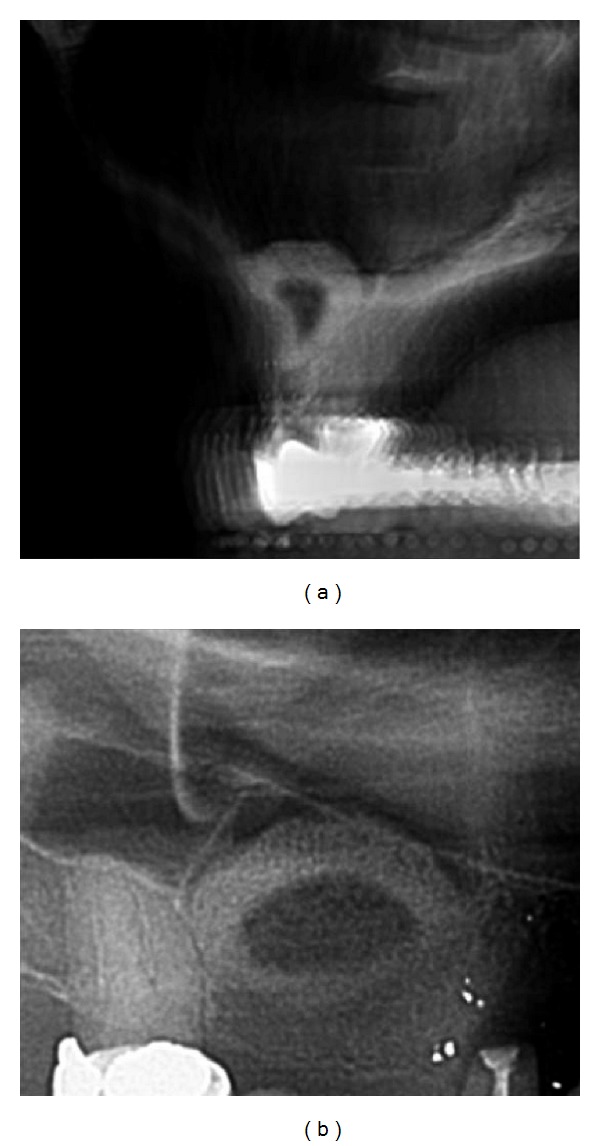
Case 3: transversal and parasagittal CBCT scan 4 months after tHUCSL-Intralift. The even circular centripetal calcification process can be observed.

A general forensic drawback in using collagenous sponges in subantral augmentation procedures might be the inability to prove the successful Sinus lift immediately after surgery since in an OPG, a radiolucent sponge can hardly be detected (Figure 8(b) and 17(b)) and only verified by the bone formation and calcification process after 3-4 months (Figures 9 and 18) or at the time of implant insertion. To establish such a subantral augmentation procedure would call for mandatory radiopaque collagenous sponges to enable radiographic verification but would possibly decrease expenses for augmentation materials.

If the reduction of absolute augmentation height of an average of 2 mm between the 4th and the 7th month subsequent surgery could be prevented by the use of calcified bone graft instead of a collagenous sponge still has to be further investigated by a similar study protocol but has to be taken into consideration in the daily routine to prevent finally insufficient augmentation heights when using radiopaque collagenous sponges. Compared to the results of the surgical technique reported by Lundgren et. al. [8] the insertion of a collagenous sponge seems to have advantages concerning more sufficient final augmentation heights.

Furthermore the results of this study suggest that after an overall period of 7 months following minimal invasive transcrestal Sinus lift, the calcification process of the augmented subantral site seems to be completed in all cases even at augmentation volumes of 2 ccm. Nevertheless this healing duration might not be applicable to lateral approach of sinus lift procedures or cases of iatrogenic puncture or minor ruptures of the sinus-membrane due to a vaster traumatization of the sinus-membrane and surgical site. This probably might result in longer bone formation and calcification duration due to healing processes and primary repair of the traumatized tissue before the bone formation and calcification process starts.

Finally the authors generally suggest to more rely on the osteogenic potential of the periosteum [4–7] and minimal invasive surgical techniques not only in Sinus lift procedures than on grafting materials of various kinds.

## Figures and Tables

**Figure 12:**
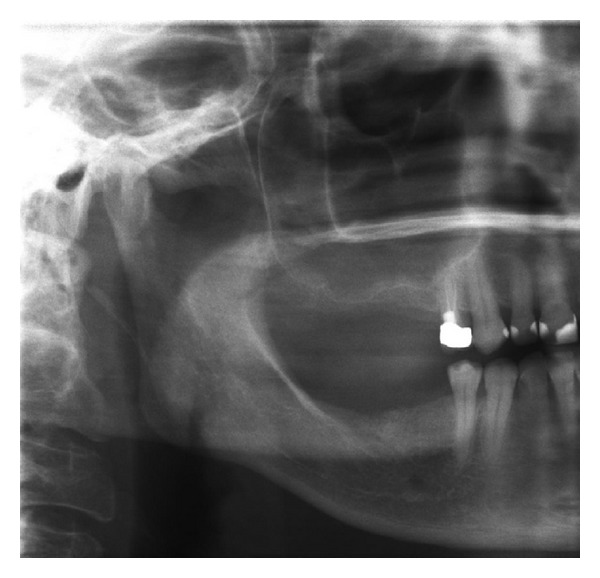
Case 2: presurgical condition in panoramic X-ray.

**Figure 13:**
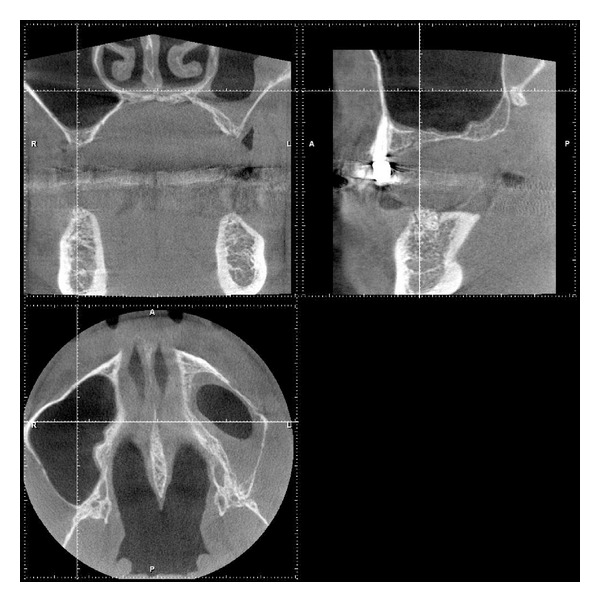
Case 2: presurgical situation in transversal, paramedian sagittal and horizontal CBCT-scan.

**Figure 14:**
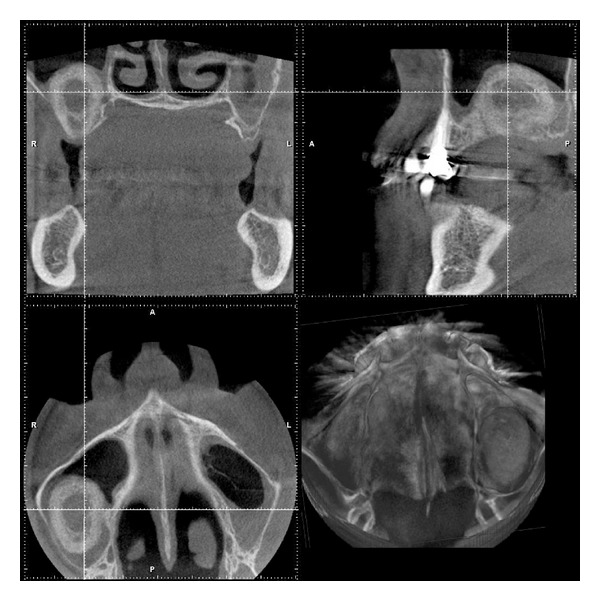
Case 2: CBCT scan 4 months following tHUCSL-Intralift. The even circular centripetal calcification process can be observed.

**Figure 15:**
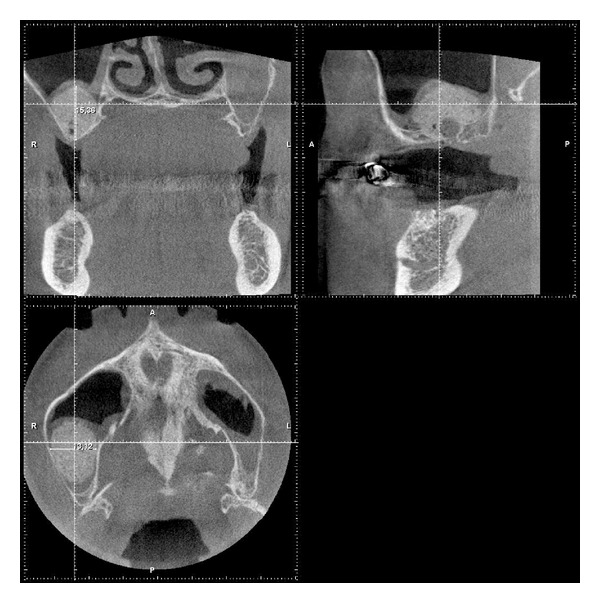
Case 2: CBCT scan 7 months after tHUCSL-Intralift: the completion of the calcification process except some smaller patches of undermineralized areas can be observed.

**Figure 16:**
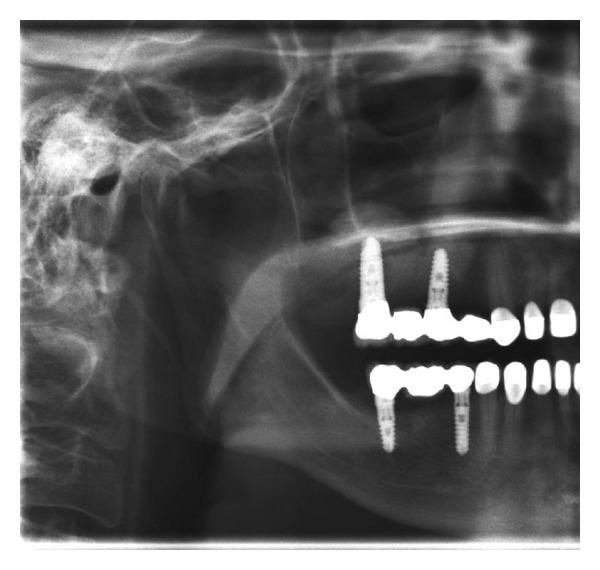
Follow-up panoramic X-ray after completion of implant insertion and prosthetic treatment 12 months following tHUCSL-Intralift.

**Figure 19:**
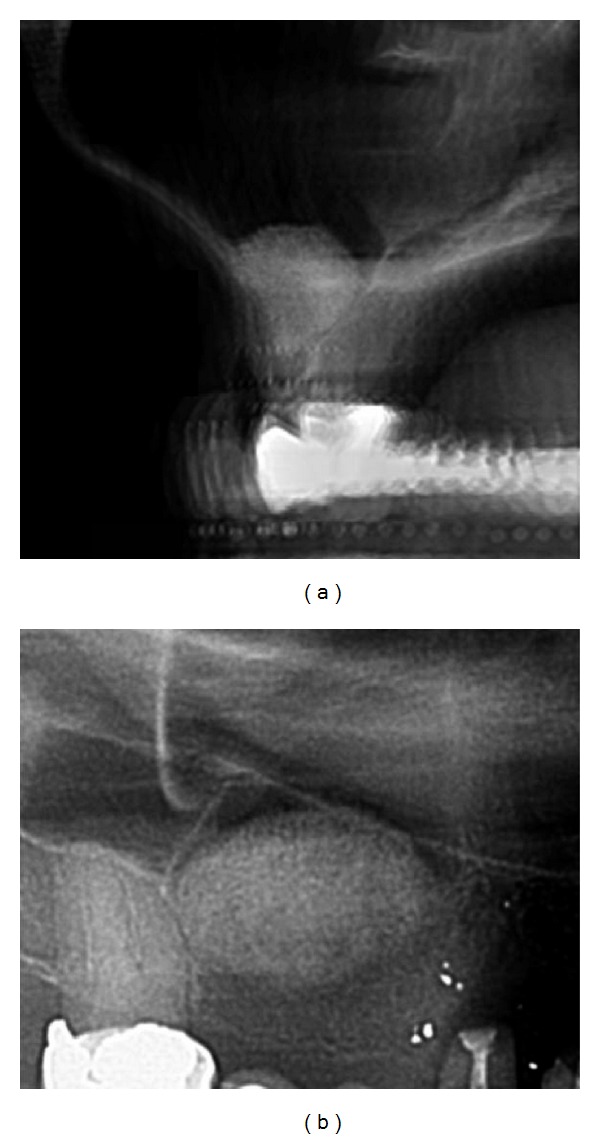
Case 3: transversal and parasagittal CBCT scan 7 months following tHUCSL-Intralift. The ossification process is obviously completed. A slim denser line on the antral floor marks the transition to the original alveolar crest.

**Figure 20:**
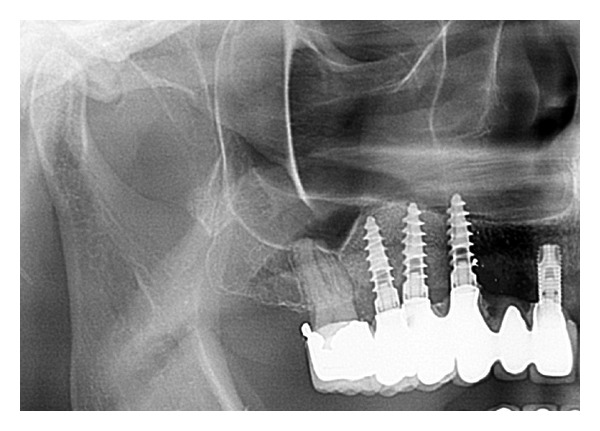
Case 3: panoramic X-ray after final prosthetic treatment after 11 months.

**Table 1:** Mean values in mm of absolute augmentation heights achieved after 4 and 7 months in sagittal and transversal CBCT slides (reference is the highest point).

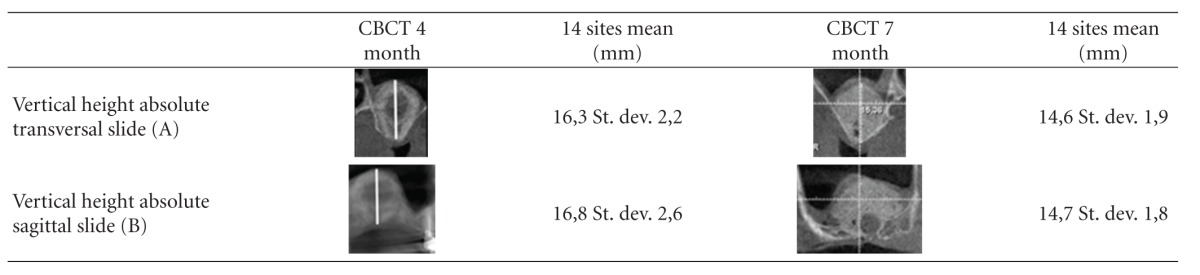

**Table 2:** Mean values in mm of calcified tissue thicknesses in 3, 6, 9, and 12 o'clock position in CBCT scans after 4 and 7 months (for reference measurement positions for transversal, sagittal, and horizontal see Figure 6).

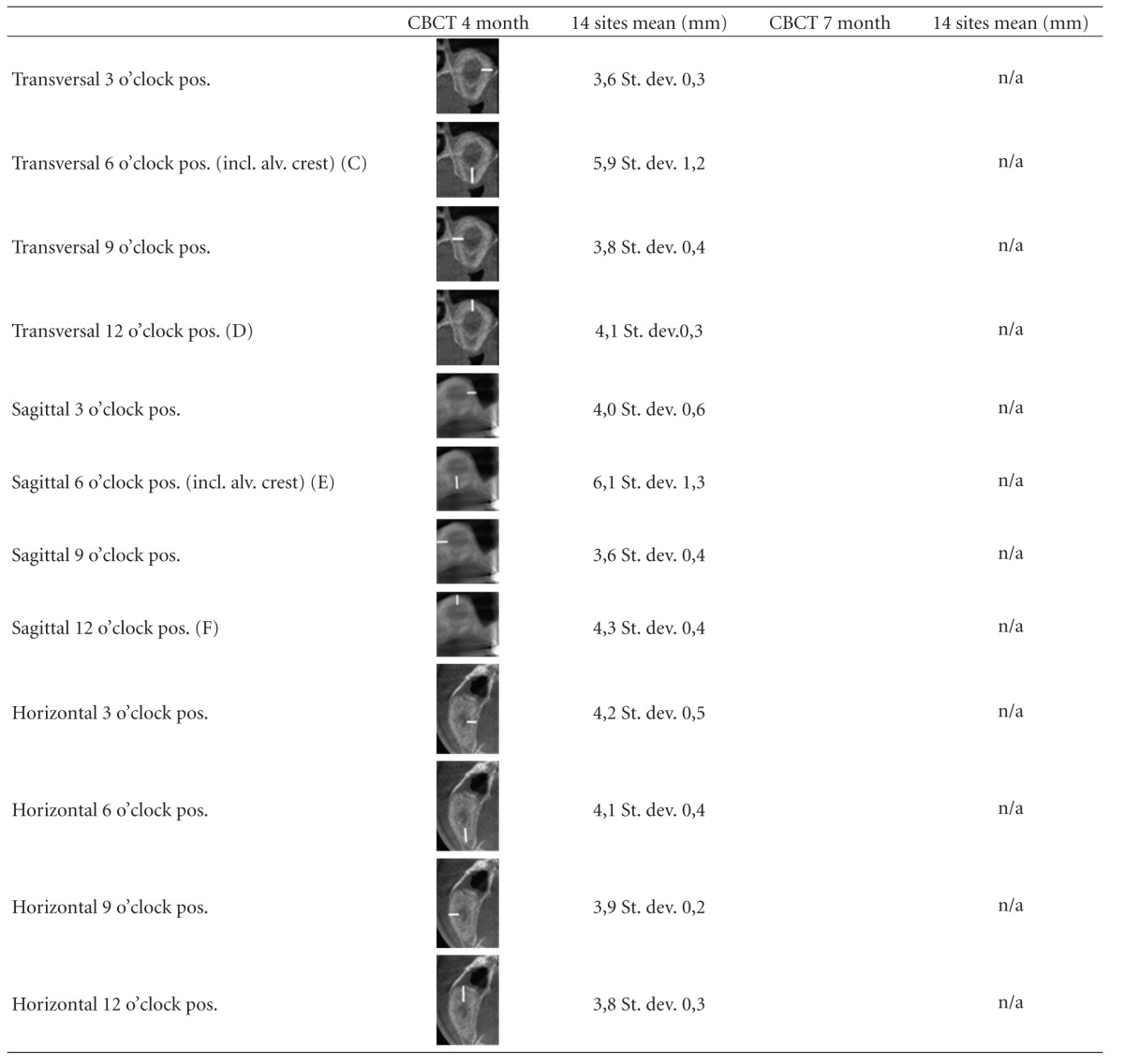

**Table 3:** Mean values in mm of absolute augmentation height loss in CBCT scans between 4 and 7 months after surgery and mean percentage of calcified tissue in 3, 6, 9, and 12 o'clock position in relation to entire distance measured (A, B ref. Table 1, C, D, E, F ref. Table 2).